# Feasibility of an online intervention (STAK-D) to promote physical activity in children with type 1 diabetes: protocol for a randomised controlled trial

**DOI:** 10.1186/s13063-016-1719-0

**Published:** 2016-12-08

**Authors:** Holly Blake, Helen Quirk, Paul Leighton, Tabitha Randell, James Greening, Boliang Guo, Cris Glazebrook

**Affiliations:** 1 University of Nottingham School of Health Sciences, A Floor, Queen’s Medical Centre, Nottingham, NG7 2HA UK; 2 University of Nottingham School of Medicine, C Floor, South Block, Queen’s Medical Centre, Nottingham, NG7 2UH UK; 3 Department of Paediatric Endocrinology and Diabetes, Nottingham Children’s Hospital, Nottingham University Hospitals NHS Trust, Nottingham, NG7 2UH UK; 4 Children’s Hospital, Leicester Royal Infirmary, Children’s Diabetes and Endocrinology, Infirmary Square, Leicester, LE1 5WW UK; 5 Institute of Mental Health, Innovation Park, Triumph Road, Nottingham, NG7 2TU UK; 6 Institute of Mental Health, B Floor, Innovation Park, Triumph Road, Nottingham, NG7 2TU UK

**Keywords:** Type 1 diabetes, Children, Self-efficacy, Physical activity, Intervention, Feasibility, Protocol

## Abstract

**Background:**

Regular physical activity has important health benefits for children with type 1 diabetes mellitus (T1DM), yet children and their parents face barriers to participation such as lack of self-efficacy or concerns around hypoglycaemia. Multimedia interventions are useful for educating children about their health and demonstrate potential to improve children’s health-related self-efficacy, but few paediatric clinics offer web-based resources as part of routine care. The Steps to Active Kids with Diabetes (STAK-D) programme is an online intervention grounded in psychological theory (social cognitive theory) and informed by extensive preliminary research. The aim of the programme is to encourage and support safe engagement with physical activity for children with T1DM. The aim of this research is to explore the feasibility of delivering the STAK-D programme to children aged 9–12 years with T1DM, and to assess the feasibility of further research to demonstrate its clinical and cost-effectiveness.

**Methods:**

Up to 50 children aged 9–12 years with T1DM and their parents will be recruited from two paediatric diabetes clinics in the UK. Child-parent dyads randomised to the intervention group will have access to the intervention website (STAK-D) and a wrist-worn activity monitor for 6 months. The feasibility of intervention and further research will be assessed by rate of recruitment, adherence, retention, data completion and adverse events. Qualitative interviews will be undertaken with a subsample of children and parents (up to 25 dyads) and health care professionals (up to 10). Health outcomes and the feasibility of outcome measurement tools will be assessed. These include self-efficacy (CSAPPA), objective physical activity, self-reported physical activity (PAQ), fear of hypoglycaemia (CHFS; PHFS), glycaemic control (HbA1c), insulin dose, Body Mass Index (BMI), health-related quality of life (CHU9D; CHQ-PF28), health service use and patient-clinician communication. Assessments will be taken at baseline (T0), 8 weeks (T1) and at 6-month follow-up (T2).

**Discussion:**

The goal of this feasibility trial is to assess the delivery of STAK-D to promote physical activity among children with T1DM, and to assess the potential for further, definitive research to demonstrate its effectiveness. Results will provide the information necessary to design a larger randomised controlled trial and maximise the recruitment rate, intervention delivery and trial retention.

**Trial registration:**

ISRCTN, ISRCTN48994721. Registered on 28 October 2016.

**Electronic supplementary material:**

The online version of this article (doi:10.1186/s13063-016-1719-0) contains supplementary material, which is available to authorized users.

## Background

Type 1 diabetes mellitus (T1DM) is one of the most common chronic diseases in childhood [1]. It is a serious illness, with rapidly increasing incidence and prevalence [1–4] and age-specific mortality double that of the general population [5]. In 2015, there were approximately 31,500 children under the age of 19 years with diabetes in the UK, the vast majority with T1DM [6]. Diabetes management is complex and costly in social, psychological and economic terms [7, 8]. Parents/carers^1^ of preadolescent children with T1DM are generally responsible for their child’s diabetes management and behaviours, making the transition from parental care to independent self-management a unique experience for children [9]. Treatment includes daily insulin injections, a healthy diet with carbohydrate monitoring and regular physical activity [10]. Poorly managed childhood diabetes can have significant lifelong consequences. Physical activity has specific benefits for diabetes control [11], reducing risk of diabetes complications and cardiovascular disease [12], reducing overweight and benefiting mental wellbeing [13]. Research has shown that children with T1DM are less active than their peers [14–17] and this can be associated with parental concerns about hypoglycaemia [18] and the exposure of chronically ill children to excessive fatigue [19].

Interventions to increase physical activity in paediatric T1DM have commonly focussed upon structured aerobic or resistance exercise training [11, 20]. These interventions work well for young people who are already active but for those children who are less active, promoting lifestyle physical activity (such as walking, active play, etc.) may be more appropriate [21]. Consequently, there is need for an intervention that encourages and supports safe engagement with physical activity for children with T1DM.

Our previous work highlights a need for theoretically-informed interventions which include psychological elements (and outcomes) [11]. Our studies have shown that parental fear of hypoglycaemia and children’s low confidence for physical activity (self-efficacy) are important barriers to being active for children with T1DM [18, 22]. Self-efficacy in particular is a critical element in increasing and sustaining physical activity levels [21, 23]. Parents have pointed to a lack of ‘digestible’ resources for physical activity promotion and health care professionals (HCPs) have similarly identified a lack of age-appropriate, evidence-based resources [24]. Consequently, intervention should be age-appropriate and build children’s self-efficacy for physical activity which is essential to enable sustained lifelong behavioural changes.

In the UK, the peak age for diagnosis of T1DM is between 10 and 14 years [25]. Interactive, multimedia interventions may be useful health promotion tools with children of this age, who are learning skills in self-managing their condition. Our previous research shows that HCPs support the concept of introducing interactive, multimedia resources focussed on behaviour change into the clinical care of children [26]. Multimedia interventions are useful for educating children about their health, demonstrate potential to improve children’s health-related self-efficacy, and could make them more able partners in face-to-face communications with HCPs [27].

Prior work has demonstrated that children with T1DM favour web-based information [28] and that they (irrespective of socioeconomic and ethnic background) are comfortable with electronic media [29]. Similarly, parents of this age group are receptive to the use of novel technologies to help manage their child’s diabetes [30]. Despite this, few paediatric clinics offer web resources for physical activity promotion as part of routine care, and technology-based interventions for children with T1DM are scarce. Existing resources are not developed for this age group; they tend to focus on ‘sport’ rather than physical activity and active lifestyles; or they focus primarily on the parent rather than the child [29, 31–35]. Computer or web-based interventions have shown to be feasible, acceptable [34, 35] and can be effective [36] in promoting physical activity in school-aged children. Consequently, there is potential for a dedicated, interactive resource specifically promoting physical activity in children with T1DM.

We have previously developed a theory-based physical activity intervention for children with chronic conditions called Steps to Active Kids (STAK) [21] which builds self-efficacy for physical activity. This includes educational materials for parents and children, a physical activity diary, group activity sessions and goal-setting strategies. This feasibility study, known as the SKIP trial (‘Supporting Kids with Diabetes in Physical activity’), will adapt and test the feasibility of these materials for use by children with T1DM [22] using an interactive, online resource called STAK-D (Steps to Active Kids with Diabetes). STAK-D is a web package delivering all aspects of STAK-D except the group activity sessions. It is intended to promote safe physical activity, build self-efficacy and reduce fear of hypoglycaemia. It includes interactive elements, including child and parent zones, physical activity tracking and goal-setting (with feedback), active role models, activity routines using video demonstrations, and a message board. It is also a medium through which parents and children can contact a diabetes professional for advice on physical activity with diabetes through an ‘Ask the Expert’ facility.

STAK-D is a complex intervention which has been designed for implementation as an adjunct to clinical care in T1DM management. It is targeted at children aged 9–12 years and their families. Targeting this age range is appropriate since participants are likely to have a level of independence commensurate with using computer-based packages at home (98%) [37] with support from their parents. Also, intervention is beneficial at this age since lifestyle behaviours which are adopted preadolescence are more likely to be sustainable and, therefore, to influence disease risk factors across the life course [38].

The STAK-D programme combines educational (information, physical activity guidance, safety information), behavioural (physical activities and activity tracking) and cognitive-behavioural (physical activity monitoring and goal-setting) strategies to promote children’s self-efficacy for physical activity. STAK-D draws upon social cognitive theory constructs of self-efficacy and observational learning [39]. The intervention targets children who have barriers to physical activity and aims to promote self-efficacy via observing role models, mastery experience and persuasion (via education). It provides general advice around regular blood glucose monitoring (e.g. before, during and after physical activities and regularly throughout the day) and healthy eating, which has been approved by diabetes specialists, but it does not give guidance on insulin dosage and administration or carbohydrate counting. For this, families are encouraged to contact their clinical team, either using the ‘Ask the Expert’ element of the website or at their standard clinic appointments.

In addition to the website, STAK-D utilises a wrist-worn activity monitor (PolarActive; Polar Electro Inc., Lake Success, NY, USA) to encourage activity monitoring and goal-setting behaviours. The site is password-protected and can currently only be accessed by research participants and personnel.

### Rationale

As there is no published data for the efficacy of a theory-based, online physical activity intervention for preadolescent children with T1DM, this research marks an important first stage in the development of an evidence base. This study will explore how feasible it is to deliver the STAK-D programme to promote self-efficacy for physical activity in this population, and its findings will inform the decision to run a future definitive clinical trial.

### Aim

The main aim of this study is to establish the feasibility of undertaking a definitive trial to investigate the effectiveness of STAK-D. The definitive trial will be deemed feasible if it is demonstrated that we can successfully identify, recruit and retain patients with T1DM, and the proposed study design and intervention are considered acceptable by patients, parents and clinicians.

### Study objectives

#### Primary research objective

The main objectives are to accomplish the following: (1) to estimate the likely recruitment rate of children and their parents, (2) to assess the willingness of the clinical staff to recruit participants, (3) to assess the willingness of eligible participants to be randomised, (4) to assess adherence and compliance to different elements of the intervention (including wrist-worn activity monitors), (5) to examine potential adverse effects of the intervention, (6) to test collection of health, clinical and economic outcome measures with the aim of informing the larger trial, (7) to explore reasons for loss to follow-up and (8) to access the overall acceptability of the intervention.

#### Secondary research objectives

A secondary research objective is to estimate the variability of self-efficacy for physical activity scores (e.g. pre-intervention to post-intervention change) and other outcome measures in order to inform decisions about primary and secondary outcomes for the larger-scale randomised control trial (RCT) and associated sample size calculation.

## Methods

The protocol has been prepared according to Standard Protocol Items: Recommendations for Interventional Trials-Extension for Newborns, Children, and Adolescents (SPIRIT-C) [40]. For the completed checklist, see Additional file 1.

### Study design

This study is a 6-month randomised feasibility trial. It is a two-arm, individually randomised, controlled feasibility trial comparing the use of the STAK-D programme to usual clinical care. A mixed-method process evaluation will be ongoing and explore rate of recruitment, adherence (pattern of intervention use), retention, data completion and adverse events. Qualitative interviews with key stakeholders (children, parents and HCPs) will explore satisfaction with the intervention and identify necessary improvements prior to proceeding to a definitive trial.

### Study setting

Participants will be recruited from two paediatric T1DM clinics in the UK. The intervention will be delivered via a website that can be accessed by participants remotely (at home) via multimedia devices.

### Selection of subjects

#### Inclusion criteria

Children aged 9–12 years who have been diagnosed with T1DM for at least 3 months and their parents will be eligible to take part in the study. They must be able to understand spoken and written English.

Children and parents eligible for the qualitative process evaluation will be those who have been directly involved with the intervention; eligible HCPs will have been directly involved in the clinical care of children in the intervention arm of the study and be familiar with the research processes; all must consent/assent to being involved in an interview.

#### Exclusion criteria

Children who meet at least one of the following criteria will be excluded: (1) recurrent hypoglycaemia (blood glucose level below 4 mmol/l occurring at least daily) or consultant concern indicating poorly managed diabetes (glycosylated haemoglobin (HbA1c) level >80 mmol/mol and/or testing fewer than four blood glucose levels/day on a downloadable meter) and (2) lacking the mental capacity to decide to take part in the study and to participate in it (based on the clinical team’s judgement in accordance with the Mental Capacity Act 2005 Code of Practice 2007).

### Interventions

#### Active intervention

The intervention will be delivered to participants randomised to the intervention arm by a health psychology researcher. The researcher will provide parents and children with login details for the STAK-D website, and conduct face-to-face orientation training with them to demonstrate the website components and assist them in setting physical activity goals. They will be encouraged to use the website for the duration of the study and for a minimum of 6 weeks. The researcher will provide the child with a PolarActive watch, together with verbal and written instructions for its use and guidance on how the activity data will be downloaded. All children in the intervention group will be provided with a supplementary leaflet signposting them to local events, services and facilities where they can engage in physical activity. Children will receive full access to all the intervention components for 8 weeks (Table 1). After 8 weeks until the 6-month follow-up (T1 to T2), participants will continue to have access to the website (Kid Zone and Parent Zone) and the PolarActive activity monitor, but they will no longer receive individualised feedback on their activity levels.

**Table 1:** Components, content and theoretical underpinning of the Steps to Active Kids with Diabetes (STAK-D) programme

Component	Content	Theoretical underpinning
Kid Zone (website)	Physical activity information and advice, 5-a-day activity target, activity tracking, goal-setting	Outcome expectations Persuasion (education) Self-regulation (goal-setting, self-monitoring) Mastery experience
Street dance routine (website)	28 × 10-min dance sessions gradually developing into a complete dance routine	Vicarious experience (role model) Mastery experience Social support
Goal-setting (website)	Personalised goal-setting and goal feedback from researchers via the website Identifying facilitators and barriers to behaviour change	Self-regulation (self-monitoring and goal-setting) Social support
‘Ask the Expert’ (website)	A way for children/parents to contact a health care professional with questions about physical activity with diabetes	Social support Persuasion (education)
Messaging Board (website)	An area where children/parents can post messages, comments and questions to other children/parents	Social support Vicarious experience
Physical activity monitor (PolarActive)	Physical activity and step-count monitoring/tracking	Self-regulation (self-monitoring and goal-setting) Mastery experience
Parent Zone (website)	Information and advice around physical activity	Social support Vicarious experience (role model)

#### Control group

Participants in the control group will not receive the STAK-D intervention, but will continue with usual clinical care according to that available at the site from which they were recruited. Both participating sites operate under the same general standardisation of care; they both adhere to Global IDF/ISPAD Clinical Practice Consensus Guidelines [41], NICE guidelines for clinical care of diabetes in children and young people [42], and the UK National Health Service (NHS) national diabetes best practice tariff, which specifies requirements for the service. Quantifying usual care with regards physical activity is difficult, although our previous qualitative research suggests that lifestyle physical activity advice is limited in the current clinical care of children with T1DM [18, 24]. Usual care at each site will be described as part of this feasibility study following recommendations by Erlen et al. [43]; this will allow for accurate definition of usual care in future trial design.

#### Outcome measures

Outcome measures will be completed at baseline (T0), 8 weeks (T1) and 6 months (T2).

#### Primary outcome measures

The primary outcome measures for the feasibility study are as follows:

1. Recruitment rate
  An estimate of the number of eligible patients and likely uptake rate will be assessed. The response rate for mailed invitation will be compared to clinic (face-to-face) recruitment rates. Refusal rate and reasons for nonparticipation will be assessed. This will enable a prediction of recruitment rate, number of sites and length of time needed to recruit the required number of patients for a future trial. A recruitment rate of between 25 and 40% would be considered reasonable based on our previous research [22] and similar studies [44, 45]
2. Adherence rate
  Pattern of intervention use will be monitored to assess fidelity of intervention delivery. We will explore users’ activity by website and page visits, more or less commonly used elements, and engagement with goal-setting and activity tracking. We will assess user satisfaction with STAK-D (e.g. navigation, ease of use, technical issues) at 6 months. Acceptable compliance will be defined as the child using the online resources and tracking their daily activity (using the PolarActive watch). Noncompliance will be defined as the child’s failure to access the STAK-D website and track their physical activity over the intervention period
3. Retention rate
  Retention will be defined as the number of participants completing the STAK-D programme including all scheduled follow-up data collection (T0, T1 and T2) compared to the number started. A retention rate of at least 70% at each time point would be considered feasible based on our previous research [22] and similar studies [44, 45]
4. Data completion rate
  Completion of outcome measures will be recorded at T0, T1 and T2 (complete and partial or noncompletion with reasons, including time to complete). Measures will be determined acceptable to parents and children (in terms of literacy, cognitive ability and capacity to understand) if more than 70% are fully completed (indicating quality data), and reported participant burden is minimal.
  The feasibility of gathering routinely collected clinic data from patients’ electronic diabetes record (height, weight, HbA1c level and insulin dosage) at T0, T1 and T2 will be assessed. This will allow us to ascertain the proportion of our sample with complete clinical records, and whether records will be accessible to our researchers at specified data collection time points. Feasibility of collecting observational data in this way will be determined if more than 85% of the sample have complete clinical records available
5. Adverse events
  Any adverse events experienced as a result of participation in the intervention will be recorded and evaluated. Adverse events are defined as any serious negative outcome resulting from the physical activity undertaken as part of the STAK-D programme. Severe hypoglycaemia, i.e., a blood glucose level below 4 mmol/l requiring the help of another person to treat it, will be considered an adverse event in either group. Data from meter and pump downloads combined (and meter downloads alone for those patients not on insulin pump therapy) will be used for identification of hypoglycaemic episodes, as per usual care at our participating sites. To determine the likelihood of adverse events being associated with the physical activity undertaken as part of the STAK-D programme, we will record whether the frequency and/or severity of hypoglycaemic episodes increased during the intervention period. The occurrence of adverse events related to the intervention will be assessed and compared to the control group (see ‘Harms’ section for potential adverse events).

#### Qualitative interviews

At the 6-month follow-up (T2), child-parent dyads in the intervention group and HCPs will be interviewed to assess their experience of participating in the trial, the acceptability of the intervention and their overall experience. All participants who drop out will be approached for an interview. The interviews will explore: (1) the willingness of participants to be randomised, (2) whether children enjoyed the STAK-D programme or not, and why, including how it made children feel, (3) children’s satisfaction with the wrist-worn activity monitor (PolarActive), (4) barriers to participation in the STAK-D programme, including reasons for nonadherence, (5) acceptability of the research processes including reasons for attrition and missing data, (6) whether parents and children thought that the intervention was beneficial, useful and easy to incorporate into daily routine, (7) children’s and parents’ perceived reasons for, and response to, any changes in outcomes, (8) willingness of HCPs to be involved in recruitment, (9) willingness of HCPs to receive and reply to emails from STAK-D programme users (via the ‘Ask the Expert’ section on the website), (10) suitability of the clinic setting for recruitment and (11) whether the inclusion criteria were deemed appropriate by patients, parents and HCPs.

#### Health care costs

Estimated costs relevant to recruitment, screening, intervention implementation and follow-up will be calculated. We will also estimate retrospectively the health care resource use and cost them at national rates.

### Secondary outcome measures

The following standardised measures have adequate psychometric properties and have been used in other studies with children of the same age range who have T1DM [22] or other long-term conditions [21]. Our previous research [22] and significant patient and public involvement activities have indicated that the assessments may take approximately 45–60 min to complete. The outcomes measured at T0, T1 and T2 will be:

#### Child measures

I. Self-reported physical activity
  Self-reported frequency of different types of physical and sedentary activities will be assessed with a 55-item physical activity questionnaire (PAQ) [46] adapted from the full 80-item PAQ [47]. The questionnaire refers to the previous 24 h and children are asked to rate a range of activities, on a three-point scale (none, a little, a lot) at three time points (yesterday afternoon, last night, this morning). Higher scores indicate greater frequency of engagement in physical activity. The authors of the PAQ reported good agreement between questionnaire responses for PAQ and observed activities [47]. Furthermore, combining interview technique with probing activity in the recent past is considered most likely to generate results that correlate with objective measures of activity [48]. The 55-item PAQ has been found to discriminate physical activity levels in children attending paediatric outpatient clinics [46]
II. Self-efficacy for physical activity
  Children’s self-efficacy for physical activity will be measured using the Children’s Self-Perceptions of Adequacy in, and Predilection for, Physical Activity scale (CSAPPA) [49]. CSAPPA is a 20-item scale to measure the self-perception of confidence in, preference for, and enjoyment of physical activity. CSAPPA is composed of three subscales: (1) perceived adequacy (perceived ability to achieve some level of successful with respect to being physically active), (2) predilection toward physical activity (preference for being active over being sedentary when given the choice) and (3) enjoyment of physical education class. The scale employs a structured alternative choice format. Children are asked to choose the option that best describes them from pairs of statements about other children such as, ‘Some kids can’t wait to play active games after school’ and ‘Other kids would rather do something else’ by indicating whether the sentence was either ‘sort of true for me’ or ‘really true for me’. Higher scores for subscales indicate greater perceived adequacy, predilection for, and enjoyment of physical activity. The scale was designed by Hay (1992) [49] for 9–16 year-olds and has demonstrated high test-retest reliability and strong predictive and construct validity
III. Fear of hypoglycaemia
  Children’s fear of hypoglycaemia will be assessed using the Child Hypoglycemia Fear Survey (CHFS) [50]. CHFS is comprised of a 10-item behaviour (B) subscale and a 15-item worry (W) subscale. HFS-B items describe behaviours performed in order to avoid hypoglycaemic episodes and/or their negative consequences (e.g. by limiting exercise or physical activity). HFS-W items ask about specific concerns about hypoglycaemic episodes (e.g. episodes occurring during sleep, or having an accident). Higher scores indicate greater fear of hypoglycaemia. Previous research has indicated that the children’s HFS is a valid and reliable measure of youth fear of hypoglycaemia and that it significantly correlated with other measures of anxiety [50]
IV. Health-related quality of life
  Child Health Utility 9D (CHU9D) [51] is a health-related quality of life measure for children aged 7–17 years, which allows the calculation of quality-adjusted life years (QALYs) for use in cost-utility analysis. It consists of nine items (worry, sadness, pain, tiredness, annoyance, school, sleep, daily routine and activities) each represented by a single question with five response options (scored 1–5). Higher scores indicate better health-related quality of life. The recall period is today/last night. The CHU9D has demonstrated validity in paediatric clinical populations [52]
V. Communication questionnaire
  A short questionnaire developed specifically for this study will be used to explore children’s perceptions of the communication around physical activity they have with their diabetes team. It contains two items and assesses children’s perceptions of (1) frequency of physical activity discussion (e.g. ‘In the last 6 months, have you spoken to one of your diabetes doctors or nurses about physical activity, exercise or sport?’ and, ‘If yes, how many times (1–5+)?’) and (2) difficulty of physical activity discussions (e.g. ‘talking to your diabetes doctors and nurses about physical activity, exercise or sport is…..?’ (5-point scale; really hard to really easy))
VI. Objective physical activity
  Physical activity will be measured objectively using a PolarActive wrist-worn activity watch (Polar Electro Inc., Lake Success, NY, USA) at T0, T1 and T2 in intervention and control group children. Wrist-worn activity monitors have demonstrated acceptability and feasibility among children with T1DM in previous research [22]. The PolarActive device has shown to be preferred by children aged 7–10 years compared with other devices, and is associated with the highest level of compliance [53]. Children will be given the PolarActive watch for 7 days at each time point, and start date (day of the week) will vary according to recruitment date; we will monitor patterns of physical activity over the data collection period. Day 1 will be considered as a familiarisation day to minimise potential reactivity and data will be analysed if activity watches are worn for at least 600 min on at least 3 days. Physical activity will be measured as step counts and average minutes in moderate-to-vigorous physical activity per day
VII. Clinical outcome measures
  These measures are routinely collected by the diabetes health care team and, with consent, will be taken from the child’s clinic notes. The measurement that coincides most accurately with the data collection period (T0, T1 and T2) will be taken.
  - *Glycosylated haemoglobin (HbA1c)* – as an indication of diabetes control
  - *Insulin dosage –* to explore changes as a further indicator of diabetes control. Insulin dosage is downloaded at standard clinic appointments using diabetes management software (e.g. Diasend/Carelink Diabetes Management); we will assess the feasibility of collecting this information from routine clinic notes. Total daily insulin dose will be recorded where possible, and we will examine the practicality of collecting this data, and subdividing it into basal and bolus doses
  - *Body composition* – Body Mass Index (BMI) will be calculated from height (cm) and weight (kg)

The primary outcome to be used in a future large-scale RCT is projected to be a change in self-efficacy for physical activity as measured by the CSAPPA scale. However, the choice of additional primary outcome measures will be finalised after taking into account the results of the feasibility study.

#### Parent measures

I. Participant characteristics
  Parent’s demographic questionnaire will assess ethnic background, family composition, parent(s) education and parent(s) occupation. Date of the child’s T1DM diagnosis, insulin delivery method and method of glucose monitoring will also be collected
II. Fear of hypoglycaemia
  Parental fear of hypoglycaemia will be assessed using the Parent Hypoglycemia Fear Survey (PHFS) [50]. The PHFS is comprised of a 10-item behaviour (B) subscale and a 15-item worry (W) subscale. Higher scores indicate greater fear of hypoglycaemia. This scale is the same as the children’s version, the only difference is that it asks about parental fear of hypoglycaemia. Research shows that the PHFS can provide reliable self-report of parental fear of hypoglycaemia [50]. It also asks parents to report the number of severe hypoglycaemic episodes that their child has experienced in the past 12 months
III. Child health-related quality of life
  This 28-item questionnaire (CHQ-PF28) assesses physical and psychosocial wellbeing of the child, from parent perspective, and can be used as a proxy quality of life measure for health-utility [54]. Higher scores indicate better health-related quality of life. The CHQ-PF28 has demonstrated reliability and validity in parents of children aged 4–13 years [54]
IV. Additional self-reported items
  Additional items self-reported by parents (‘follow-up questionnaire’) will include: (1) number of additional contacts with the diabetes team other than routine clinic visits in the last 6 months, (2) number of hospital admissions other than routine clinic visits in the last 6 months, (3) days off school in the last 6 months, (4) additional medications in the last 6 months and (5) perceived frequency of communication about physical activity in consultations with the diabetes health care team

#### HCP measures

I. Clinician-patient communication
  A nine-item clinician-patient communication questionnaire has been developed specifically for this study and will be used to explore HCPs’ perceptions of the communication around physical activity they have with their patients. It aims to assess (1) how often clinicians have discussed physical activity with their patients and (2) how clinicians feel about talking about physical activity based on how much they agree with statements (e.g. ‘I am able to talk to children and their parents about physical activity.’)

#### Participant timeline

**Fig. 1:**
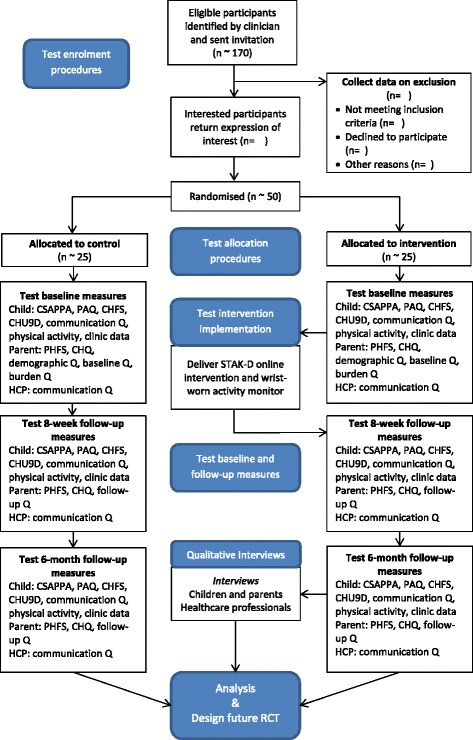
Flow diagram of the study. *CHFS* Child Hypoglycaemia Fear Survey, *CHQ C*hild Health Questionnaire, *CHU9D* Child Health Utility Instrument, *CSAPPA* Children’s Self-Perceptions of Adequacy in, and Predilection for, Physical Activity scale, *HCP* health care professional, *PAQ* Physical Activity Questionnaire, *PHFS* Parent Hypoglycaemia Fear Survey, *Q* questionnaire, *RCT* randomised controlled trial, *STAK-D* Steps to Active Kids with Diabetes

Figure 1 shows a flow chart of study processes. Table 2 shows the assessments at each time point.

**Table 2:**
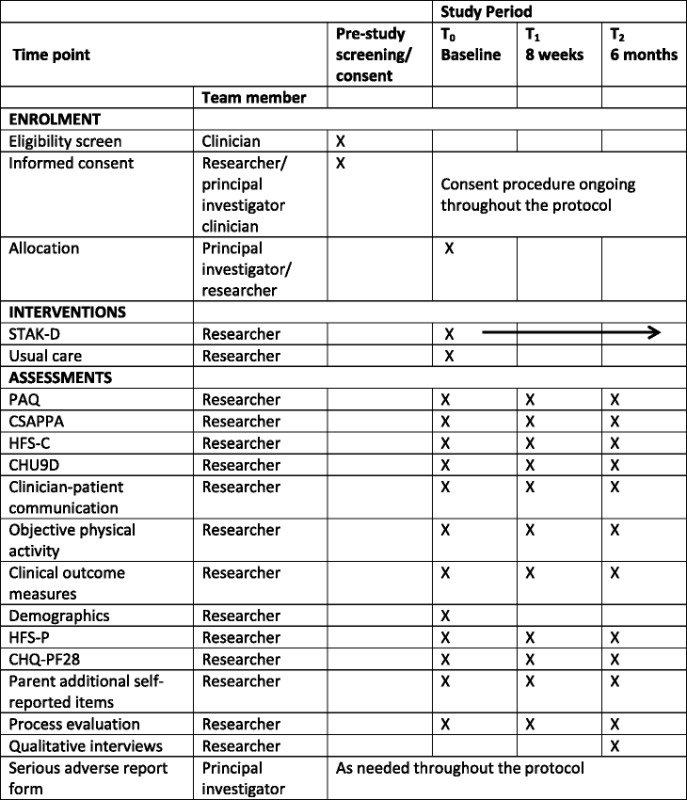
Study assessments at specific time points

### Sample size

The sample size will be adequate to estimate the critical parameters (e.g. recruitment rate) to the necessary degree of precision and to provide key information on the feasibility of the intervention. There are approximately 170 patients in the specified age range across both recruitment sites, prior to exclusions. We will target the recruitment of 50 child-parent dyads (approximately five per month over 9–10 months). We envisage this to be achievable since previous studies indicate a 40–50% response rate with similar populations [22, 55] and our selected feasibility study sites currently have recruitment rates exceeding 100% for NIHR-adopted studies. Recruitment of a minimum of 33 child-parent dyads (40%) would be deemed necessary to progress to full trial; should recruitment be problematic a remedial act will be to revise inclusion criteria to include age range 8–13 years. At T2, up to 25 child-parent dyads and up to 10 HCPs will take part in interviews.

### Recruitment

Potential participants will be recruited from paediatric diabetes clinics at the two hospital sites. They will be identified and approached by a member of the clinical team via a postal invitation pack (including letter, Participant Information Sheets (PIS) and Expression of Interest (EOI) Form). Children will receive a child-friendly version of the PIS. If the child and parent are willing to hear more about the study, they will return the EOI to the research team, and will be introduced to the researcher at their next clinic appointment for a detailed discussion about the study. At this prestudy screening meeting, consent will be received, baseline data collected and randomisation performed. Participants will be randomised after baseline data has been collected. The researcher and/or principal investigator will receive written informed consent from the parent and written informed assent from the child prior to the participant undergoing any research procedures. There must be mutual agreement between the child and parent for the child to participate.

### Assignment of interventions

#### Sequence generation

Consented participants will be individually randomised with a 1:1 allocation to receive either the intervention or usual care; no participant will receive less than standard care. The allocated treatment will be determined using the ‘Sealed Envelope Company’ randomisation service [56]. After receiving consent and assent, and collection of baseline data, the researcher will send an SMS text message to the ‘Sealed Envelope Company’, which will automatically randomise the participant to the intervention or control group.

#### Blinding

Neither participants nor the researchers delivering the intervention can be blinded due to the nature of the intervention. Wherever possible, the data analysts (e.g. statistician) will be blinded to treatment allocation. The follow-up assessor may not be blinded given resource constraints.

### Data collection, management and analysis

#### Data collection methods

Participants will have six visits for data collection. Visits will take place at either the child’s usual paediatric diabetes clinic or another mutually convenient location (e.g. the participants’ home) depending on parent preference, and preferred locations for data collection will be recorded. Children and parents will complete the assessments at visits 1 (baseline/T0), 3 (T2) and 5 (T3). Children will be assessed for self-reported physical activity (PAQ), self-efficacy for physical activity (CSAPPA scale), fear of hypoglycaemia (CHFS), health-related quality of life (CHU9D), clinician-patient communication and objective physical activity (PolarActive). As close to visits 1, 3 and 5 as possible, the researcher will ask the clinician for clinical data from the participants’ clinical records (height, weight, HbA1c level and insulin dosage). Parents will be assessed for demographics, fear of hypoglycaemia (PHFS), child’s quality of life (CHQ-PF28) and additional self-reported items. One week after visits 1, 3 and 5 (at visits 2, 4 and 6), data from the activity watch will be downloaded. Health care professionals drawn from the paediatric diabetes teams at the study sites will be asked to complete a clinician-patient communication questionnaire at T0, T1 and T2.

At visit six, all child-parent dyads in the intervention group will be invited to participate in single exploratory interview as part of the process evaluation. All participant dyads who have dropped out of the study will be invited to interview (at the point when they leave the study). For practical reasons child and parent will be interviewed together with questions/topics directed to each as appropriate. Interviews will be undertaken face-to-face at a time and venue convenient to the family.

Up to 10 HCPs who have been directly involved in the care of children who have received the intervention (including all those listed on each site file delegate log) will be interviewed at the end of the study to gain their perspective upon STAK-D delivery and the research processes. Interviews with HCPs will be carried out in person or over the telephone and will be recorded, with permission from the interviewee. In particular, their willingness to identify and approach patients, their opinion about patient randomisation, and their comments upon delivering and supporting STAK-D (specifically whether they referred to STAK-D in their consultations) will be considered. During these interviews the integration of STAK-D with clinical practice and other NHS systems will be explored. Interviewees will be drawn from the paediatric diabetes teams at the study sites, and the sample will be constructed to reflect the range of professions that is involved the care of children with T1DM who have been exposed to the intervention. Wherever possible, individuals refusing to participate in the study will be asked their reason(s) for nonparticipation.

### Data management

Standard procedures following the Data Protection Act (1998), the NHS Code of Confidentiality, and Good Clinical Practice (GCP) will be implemented throughout the study.

#### Statistical methods

##### Primary endpoint: feasibility

Descriptive statistics will be used to describe the sample characteristics and rates of recruitment, retention, completion and adherence (frequencies, percentages, means and standard deviations or medians and interquartile range). Adverse events will be reported descriptively.

All interview data will be recorded digitally, transcribed in full, anonymised, and handled using the NVivo software package (QSR International Pty Ltd., 2014). Following the conventions of framework analysis [57] a pragmatic analytic framework will be constructed and all interview data charted against this. The framework will consist of two analytic matrices (one to address STAK-D delivery, one to address trial design) which will be structured to aid identification of elements which worked well and those which require further adaptation prior to a larger clinical trial. Interviews with parent(s), children and HCPs will be analysed using thematic analysis. A minimum of two random transcripts will be re-coded by an independent researcher to ensure consistency.

##### Secondary endpoint: effect

Effectiveness outcomes will be described at each time point using descriptive and inferential methods for categorical, continuous and/or ordinal health outcome measures using an intention-to-treat approach, although imputation of missing outcome data will not be performed for the primary analysis as this is a feasibility study. Reasons for missing data will be documented, and missing data will be quantified. Inferential analysis of outcomes will be presented as 95% confidence intervals. Exploratory modelling will be used to investigate factors found to be, or assumed to be, related to intervention effectiveness outcomes. Data will be analysed using the Statistical Package for Social Sciences (SPSS Inc., Chicago, IL, USA) and Stata (StataCorp, College Station, TX, USA). The level of statistical significance will be set at 5% for the primary outcome measures. A detailed Statistical Analysis Plan will be written by the statistician, in consultation with the research team, prior to analysis. Continuous variables will be reported by descriptive statistics (nonmissing sample size, mean, standard deviation, median, maximum and minimum). Categorical variables will be summarised using frequencies and percentages. The descriptive statistics from the selected primary outcomes will inform the sample size calculation of the main RCT.

#### Monitoring

##### Data monitoring

Given the small scale of this feasibility trial and the low risk of harm, an external Data Monitoring Committee (DMC) will not be needed and an interim analysis will not be performed. The researchers recruiting, implementing, and assessing the intervention will update the research team monthly about the study progress.

##### Auditing

Monitoring of trial data will include confirmation of informed consent; source data verification; data storage and data transfer procedures; local quality control checks and procedures, back-up and disaster recovery of any local databases and validation of data manipulation. The chief investigator, or where required, a nominated designee of the sponsor, will carry out monitoring of trial data as an ongoing activity. Entries on Case Report Forms (CRFs) will be verified by inspection against the source data. A sample of CRFs (10% or as per the study risk assessment) will be checked on a regular basis for verification of all entries made. In addition the subsequent capture of the data on the trial database will be checked. Where corrections are required these will carry a full audit trail and justification.

##### Harms

A side effect of physical activity for people with T1DM is changes in blood glucose levels, above or below normal, causing unfavourable symptoms. Hypoglycaemia is when blood glucose levels drop too low and hyperglycaemia is where blood glucose levels spike too high. The hypoglycaemic and hyperglycaemic risks in this research are no more severe than expected for the participant’s condition (e.g. as a side effect of insulin treatment). Furthermore, the physical activity encouraged through this intervention is not of a high enough intensity or a long enough duration to be deemed dangerous or high-risk to children (the proposed modifications to physical activity levels are in accordance with published guidelines for paediatric T1DM [42]). However, if there is considerable exacerbation (increase in occurrences or severity compared to before participation in the intervention) of episodes of hypoglycaemia and/or hyperglycaemia (blood glucose >14 mmol/l), then the participant will be asked to discontinue their use of the intervention, re-establish their previous daily routine and seek medical advice.

Adverse events will be classified on the basis of severe hypoglycaemia or hyperglycaemia, or any other injury or incident believed to be caused by participation in the STAK-D intervention. All information pertaining to adverse events noted by the researcher during the study will be listed by subject, detailing the episode date and time of onset and date and time of resolution. The onset of adverse events will be shown relative (in number of min/h/days) to the time of the highest bout of physical activity.

##### Dissemination

A research paper will report the primary outcome measures. The results will be disseminated regardless of the magnitude or direction of effect. The study results will also be disseminated to the clinical teams in the participating centres and to the participants.

#### Post-trial care

##### Archiving

Study-related documents will be archived at the lead institution, on behalf of the NHS trust study sponsor, at the end of the study for at least 10 years and in line with all relevant legal and statutory requirements.

#### Roles and responsibilities

##### Trial Management Group

The sponsor is the lead organisation where the research is to take place; Nottingham University Hospitals (NUH) NHS Trust will act as sponsor for this study (NUH NHS Trust). NUH NHS Trust will act as guarantor for the research, ensuring that it complies with standards of GCP. The chief investigator (HB) has overall responsibility for the study, supported by CG and all other team members. An appointed researcher will be responsible for the daily monitoring and management, reporting directly to HB. The two study site principal investigators (TR, JG) will oversee the identification of potential participants as well as providing expert advice during the study and the analysis and interpretation of the results. BG is the study statistician.

## Discussion

Among physical activity and exercise interventions for young people with T1DM to date, most have neglected lifestyle physical activity in favour of structured aerobic or resistance exercise training, few have been underpinned by psychological theory of behaviour change, little attention has been given to potential psychological outcomes and technology-based interventions have been scarce [11]. The current feasibility trial will provide data to inform a larger trial, if required, to test whether the STAK-D programme can promote self-efficacy for physical activity in children with T1DM.

The study aims to help children with T1DM and their families, and ultimately to reduce NHS costs, through possible reductions in the use of NHS services in the longer term as children with T1DM will lead a healthier, more active lifestyle and have better managed diabetes in the long term. If feasible, we hope that STAK-D will be disseminated nationally as a low-cost physical activity promotion programme to support self-management of paediatric T1DM.

### Trial status

The trial was proposed at the time of original submission. Recruitment is ongoing at the time of revisions being submitted.

## Additional file

Additional file 1: SPIRIT Checklist. (DOC 127 kb)

## Abbreviations

CHFS: Child Hypoglycaemia Fear Survey
CHQ: Child Health Questionnaire
CHU9D: Child Health Utility 9D Instrument
CRF: Case Report Form
CSAPPA: Children's Self-Perceptions of Adequacy in, and Predilection for, Physical Activity scale
HCP: Health care professional
PAQ: Physical Activity Questionnaire
PHFS: Parent Hypoglycaemia Fear Survey
PIS: Participant Information Sheet
RCT: Randomised controlled trial
REC: Research Ethics Committee
STAK-D: Steps to Active Kids with Diabetes
T1DM: Type 1 diabetes mellitus

## Footnotes

1 Reference to parent refers to parent and/or carer throughout.
